# Music performance anxiety: development and validation of the Portuguese music performance anxiety scale

**DOI:** 10.3389/fpsyg.2024.1436216

**Published:** 2024-07-12

**Authors:** Samuel Barros, Alex França, Helena Marinho, Anabela Pereira

**Affiliations:** ^1^Department of Communication and Arts, INET-md, University of Aveiro, Aveiro, Portugal; ^2^Psicometria Online Academy, São Paulo, Brazil; ^3^Department of Psychology, CIEP, University of Évora, Évora, Portugal

**Keywords:** music performance anxiety, music performance anxiety scale, Portuguese higher music education, musical performance, scale development, health, wellbeing, Portugal

## Abstract

Several studies have developed and validated specific scales to understand, identify and confirm research hypotheses associated with music performance anxiety (MPA). These scales mostly assess behavioral, cognitive, and physiological factors. There is currently no original MPA assessment tool for higher music education in Continental Portuguese, which suggests a research gap. The aim of this study was to determine if the Portuguese Music Performance Anxiety Scale (PoMPAS), developed for this research, is a valid and reliable measure of MPA for the context of higher education in Portugal. The total sample was *N* = 414 (166 male, 245 female, and three without gender identification). The development of this scale was based on a three-dimensional model (behavioral, cognitive, and physiological), following the theoretical models of Salmon (1990) and Osborne and Kenny (2005). Confirmatory factor analysis of the PoMPAS suggested a good fit in a three-dimensional model with 27 items. The internal consistency values proved appropriate, showing good Cronbach’s alphas (between *α* = 0.81 and *α* = 0.90). The McDonald’s Omega also demonstrated good consistency (between *ω* = 0.81 and *ω* = 0.90). The PoMPAS is a reliable tool to measure the impact of MPA, with good psychometric qualities, specifically for the Portuguese higher music education context.

## 1 Introduction

Music performance anxiety (MPA) affects musicians of all ages, regardless of experience or level of proficiency: it is reported by musicians as the fear of failure and/or fear of negative public evaluation, and is perceived as a real threat, which triggers behavioral, cognitive and physiological responses (Kenny, 2011). For Kenny (2011), MPA is a complex phenomenon caused by the interaction of many factors: genetics, contextual stimuli, musical experience, emotions, cognitions and individual behaviors. Likewise, in the context of higher music education, a systematic review by Barros et al. (2022) highlighted several predictors of MPA or risk factors associated with a pronounced occurrence of MPA, including situational factors, social perception, individual variables, psychophysiological symptoms, gender, performance experience and age, and institutional culture.

As suggested by recent research, the higher education context evidences a high prevalence of MPA, ranging between 16% (Paliaukiene et al., 2018), 39% (Zarza et al., 2016b), 75.1% (Bannai et al., 2016) and 83.1% (Miller and Chesky, 2004). Although the results of these four studies cannot be generalized and the studies took place in different countries (Lithuania, Spain, Japan and the United States, respectively), these percentages are a warning sign for educational institutions because, if left unaddressed, MPA levels may tend to transfer to the professional sphere and even cause career dropout (Orejudo et al., 2018; Wang and Yang, 2024). A systematic review by Fernholz et al. (2019) analyzed 43 studies and stated that the prevalence in the professional context ranges from 16.5 to 60%. The comparison between prevalences suggests that MPA levels are higher among music students in higher education than among professional musicians.

According to Barlow’s theory (Barlow, 2000), the development of anxiety disorders is characterized by three types of vulnerabilities: (1) generalized biological vulnerability, which suggests that the development of anxiety is connected to a genetic contribution; (2) generalized psychological vulnerability, indicating that early experiences, under specific conditions, contribute to the development of psychological vulnerabilities and predisposition to a negative state in general; and (3) specific psychological vulnerability, where anxiety is associated with early learning experiences that occurred in some life circumstances, such as in a social assessment, which may increase the perception of threat or danger.

Supported by Barlow’s theory, Kenny (2011) highlights three subtypes of MPA: (i) MPA as focal anxiety, in which there is no generalized social anxiety; (ii) MPA that co-occurs alongside with other manifestations of social anxiety; and (iii) MPA that co-occurs with panic and depression. These perspectives suggest that MPA may be linked to an intersection between the individual’s developmental history (which may be more or less impactful in the case of focal MPA and more severe in the third subtype) and specific psychosocial conditions (such as performance requirements, technical and musical preparation, public exposure or competitiveness). Acknowledging this intersection, several researchers (Kreutz et al., 2008; Ginsborg et al., 2009) have recommended preventive health programs for musicians, designed to increase the quality of musical performance and students’ health and well-being. Nonetheless, according to research (Papageorgi et al., 2010; Zarza et al., 2016a; Casanova et al., 2018), an understanding of the context of the performance situation (e.g., playing solo or in a group, the performance environment, competition) is required to recognize and prevent MPA. Hence, the development of reliable psychometric instruments can contribute to identify contextual specificities of MPA.

### 1.1 Psychometric instruments of MPA

Several studies have applied and/or developed and validated inventories and scales to understand, identify and confirm hypotheses associated with MPA, as regards behavioral, cognitive, and physiological responses. However, none of the instruments presented below analyze or evaluate the context of the performance situation, consequently limiting the understanding of MPA in varied contexts.

The inventories commonly used in studies addressing MPA include: (i) the Music Performance Anxiety Scale (MPAS, Wolfe, 1989): 55 items addressing adaptive and maladaptive anxiety, MPA, cognitive and emotional components; (ii) the Music Performance Anxiety Questionnaire (MPAQ, Lehrer et al., 1990): 32 items concerning coping with anxiety, judgmental thoughts about performance, worrying about the impact of anxiety on performance, and concerns with the reaction of others, oneself and the audience; (iii) the Performance Anxiety Questionnaire (PAQ, Fehm and Schmidt, 2006): 20 items related to cognitive and somatic feelings and two additional qualitative items about coping strategies and performance anxiety feelings; (iv) the Kenny Music Performance Anxiety Inventory (K-MPAI, Kenny, 2009): 40 items regarding proximal somatic anxiety and worries about performance, worry/dread (negative cognitions) focused on self/other scrutiny, depression/hopelessness (psychological vulnerability), parental empathy, memory, generational transmission of anxiety, anxious apprehension and biological vulnerability; (v) the Performance Anxiety Scale for Music Students (PASMS, Cırakoğlu and Şentürk, 2013): 24 items associated with MPA, such as fear of stage, avoidance and symptoms; (vi) the Music Performance Anxiety Scale (MPAS, Sheriff and Yoong, 2015): 58 items about causes/situational factors, temporal occurrence, cognitive, affective, behavioral and somatic manifestations, and autonomic arousal; and (vii) the Mazzarolo Music Performance Anxiety Scale (M-MPAS, Mazzarolo and Schubert, 2022): 5 items to measure the global frequency and intensity of MPA episodes and their negative impact (aversion to future music performances).

Among the instruments presented above, the K-MPAI, which presents good psychometric properties, has been broadly translated and validated for other languages, including Continental Portuguese (Dias et al., 2022) and Brazilian Portuguese (Rocha et al., 2011), with a Cronbach’s alpha coefficient of *α* = 0.91 and *α* = 0.957, respectively. The four factors and 30 items of the Continental Portuguese version were extracted through an exploratory factor analysis. Nevertheless, it was not developed and designed for the Portuguese context.

It is noteworthy that Trigo (2015), in his research on the translation and validation of the MPAI-A into Portuguese – an inventory designed for samples of adolescent music students developed by Osborne and Kenny (2005), detected weaknesses concerning the principal component analysis (scale items), requiring further research. This weakness may be associated with problems in adapting this inventory to Portuguese. A scale validation study for other languages is relevant but does not take into account a contextual understanding of the specific environment (Hofmann et al., 2010; Kirmayer, 2014a,b; Rocha Zaidhaft and Ortega, 2021). Thus, there is currently no specific MPA assessment tool developed for the context of higher music education in Portugal, which suggests a significant research gap.

This research aimed to determine if the Portuguese Music Performance Anxiety Scale (PoMPAS) – in Portuguese: “Escala Portuguesa de Avaliação da Ansiedade na Performance Musical (EPAAPM)” – is a valid and reliable measure for the context of higher music education in Portugal, based on theoretical models (Salmon, 1990; Osborne and Kenny, 2005) that highlight behavioral, cognitive, and physiological dimensions. In this study, we aimed to add and evaluate the contextual factor of the performance situation, such as the formalities associated with performance, or the contexts of competitions or orchestral auditions, and the physiological symptoms experienced before and during the performance.

The research question that supports this research is: Can an inventory specifically designed for the Portuguese context adequately assess the impact of MPA in Portuguese higher music education? To answer this question, the research involved designing a scale for this context that assesses the behavioral, cognitive, and physiological responses, adding items that can also measure the impact of the performance situation and its associated physiological dimensions.

## 2 Method

### 2.1 Participants and sample characteristics

Seven hundred and three students from public and private higher education institutions (universities and polytechnic schools) participated in this study. Participants were invited through institutional emails, personally by the first author of this research, and via social networks such as Instagram, Facebook, and WhatsApp, in order to reach a wide and diverse range of participants and institutions.

The criteria for participation in this study were: (1) being enrolled in a higher education institution in Portugal and currently attending bachelor, master or PhD courses, and (2) answering all the items in both questionnaires. The higher education system in Portugal includes two main types of institutions: universities and polytechnic institutes. The universities can offer bachelor, master and PhD courses, while the polytechnic institutes usually offer only bachelor and master courses.

Although 703 students participated, we only considered valid the fully answered questionnaires. Thus, the total sample was *N* = 414 (Table 1). Of these, 166 were male (40.1%), 245 were female (59.2%), and three did not want to identify their gender (0.7%).

**Table 1:** Characterization of participants by age, gender, institution, degree, and instrument (*N* = 414).

	Min	Max	Mean	SD	*N*	%
Age	18	60	23.78	6.46	414	100
Gender	Male				166	40.1
	Female				245	59.2
	Other				3	0.7
Institution type	Public university				248	59.9
	Private university				23	5.6
	Public polytechnic				139	33.6
	Private polytechnic				4	1.0
Graduation type	Bachelor program				229	55.3
	Master program				150	36.2
	Doctoral program				35	8.5
Instrument type	Brass				89	21.5
	Woodwind				108	26.1
	Frictional strings				90	21.7
	Finger strings				28	6.8
	Keyboard				57	13.8
	Voice				30	7.2
	Electric instrument (bass)				1	0.2
	Percussion				7	1.7
	Conduction (regency)				4	1.0

The ages ranged from 18 to 60 (*M* = 23.78; *SD* = 6.46). We established *a priori* the need for a sample with an absolute minimum of 400, as it simultaneously agreed with Comrey and Lee’s guidelines (Comrey and Lee, 1992) and Nunnally’s (1978) criterion of a subject-to-item ratio of 10:1 (at least 10 participants per item).

### 2.2 Procedure

Two instruments were developed for this study.

#### 2.2.1 Sociodemographic questionnaire

A sociodemographic questionnaire was created to collect participant data (Table 1) such as age, gender, institution, graduation, and instrument.

#### 2.2.2 Portuguese music performance anxiety scale

Prior to developing the PoMPAS, we conducted a qualitative study (Barros et al., 2024), which supported the formulation of the initial questions (bank of items) associated with the context of Portuguese higher education music students, identifying latent MPA-related perceptions about symptoms and contextual factors. Thus, we planned, *a priori*, a scale with four main factors: (1) behavioral/emotional, (2) cognitive, (3) physiological/somatic, and (4) the context of the performance situation (new factor). In addition, some of the K-MPAI’s items (Kenny, 2009) were adapted as models for strengthening the creation of the bank of items of the new scale.

Separate sessions of thinking-aloud discussion, with music students (eight), music researchers (two) and psychology researchers (two), following Van Someren et al. (1994), were undertaken to verify the semantic content and assessing its appropriateness.

The preliminary version of the PoMPAS included 40 items. A Likert scale with five response options was used, with scores from 1 (I completely disagree) to 5 (I completely agree).

The sociodemographic questionnaire and the scale were available online via the LimeSurvey interface at https://forms.ua.pt/, from December 2021 to September 2022, with the ID 488167.

#### 2.2.3 Ethical consideration

We provided an informed consent form to each participant, informing about the purpose of the study and that they could withdraw at any time and without any consequences, and that all responses would be confidential and anonymous. This research furthermore complied with the General Data Protection Regulation (GDPR) of the European Union and was approved by the Ethics and Deontology Board of University of Aveiro (Certificate 02-CED/2020).

### 2.3 Data analysis

Descriptive analysis was undertaken to describe the participants’ sociodemographic characteristics (mean, frequency and percentages – Table 1). To analyze the factor structure of the PoMPAS, a psychometric study was carried out through exploratory factor analysis (EFA) and confirmatory factor analysis (CFA). The Solomon method was used to split the total sample into equivalent subsamples for exploratory and confirmatory factor analysis (Lorenzo-Seva, 2022).

#### 2.3.1 Exploratory factor analysis

The sample adequacy and factorability were assessed using the Sample Adequacy Index: Kaiser-Meyer-Olkin (KMO - > 0.70) and the significance of Bartlett’s test of sphericity. The extraction method used was Weighted Least Squares Mean and Variance Adjusted (RDWLS) (Asparouhov and Muthén, 2010) with oblique rotation using Robust Promin (Lorenzo-Seva and Ferrando, 2019). To determine the most appropriate number of factors, Parallel Analysis techniques with random permutation of observed data were employed (Timmerman and Lorenzo-Seva, 2011). Standardized factor loadings ≥0.30 were considered relevant for item retention in the model. After each item exclusion, a new factor analysis was conducted following the same procedures until the structure contained only items with factor loadings above the established threshold (≥ 0.30). The stability of factors was assessed using the H index (Ferrando and Lorenzo-Seva, 2018). The H index evaluates how well a set of items represents a common factor (Ferrando and Lorenzo-Seva, 2018). H values range from 0 to 1. High H values (> 0.80) suggest a well-defined latent variable that is more likely to be stable across different studies. Low H values suggest a poorly defined latent variable that is likely to be unstable across different studies (Ferrando and Lorenzo-Seva, 2018).

#### 2.3.2 Confirmatory factor analysis

The Diagonally Weighted Least Squares (DWLS) estimator with robust standard error calculation was used due to the ordinal nature of the Likert scale measurement (DiStefano and Morgan, 2014). The adequacy of the estimated model was evaluated using the chi-square (*χ2*), degrees of freedom (*df*), and the *χ2/df* ratio, along with fit indices such as Root Mean Square Error of Approximation (RMSEA), Comparative Fit Index (CFI), Tucker-Lewis Index (TLI), and Standard Root Mean Square Residual (SRMR). The *χ2/df* ratio should be less than five or preferably less than three, RMSEA values should be less than 0.08, and CFI and TLI values should preferably be >0.95. The Standard Root Mean Square Residual (SRMR) should be <0.08 (Hu and Bentler, 1999).

Internal consistency was assessed using Cronbach’s Alpha and McDonald’s Omega. Additionally, as evidence of score accuracy, the Average Variance Extracted (AVE) was calculated as a measure of the amount of variance captured by a construct relative to the amount of variance due to measurement error. Cronbach’s alpha and McDonald’s omega were calculated for Exploratory and Confirmatory analysis. Values ≥0.70 were considered adequate.

Exploratory Factor Analyses were conducted using FACTOR software version 12.04.05 (Ferrando and Lorenzo-Seva, 2017). Confirmatory Factor Analyses and internal consistency analysis, via Cronbach’s Alpha and McDonald’s Omega, were performed using JASP software (version 0.18.03).

## 3 Results

### 3.1 Exploratory factor analysis

An exploratory factor analysis was carried out on the preliminary 40-item PoMPAS. Regarding the sample, a ratio of five respondents per estimated item was obtained (Watkins, 2018). The dataset was initially analyzed to detect inconsistent values related to participants’ responses to the instrument’s items. No inconsistencies or missing data were detected. Table 2 provides a univariate descriptive analysis for the 40 items of the PoMPAS.

**Table 2:** Univariate descriptive analysis for the items of the PoMPAS.

Item	Mean	Confidence Interval (95%)	Variance	Skewness	Kurtosis (Zero centered)
1	2,705	(2.49 2.92)	1,483	0.273	−0.865
2	3,150	(2.92 3.38)	1,635	−0.157	−1,056
3	4,010	(3.82 4.20)	1,150	−0.823	−0.246
4	3,928	(3.72 4.14)	1,381	−0.917	−0.168
5	2,836	(2.62 3.05)	1,500	0.190	−0.822
6	3,343	(3.10 3.59)	1887	−0.321	−1,120
7	3,101	(2.85 3.36)	2043	−0.120	−1,246
8	2,696	(2.45 2.95)	1961	0.257	−1,254
9	3,208	(2.97 3.45)	1846	−0.126	−1,208
10	3,478	(3.26 3.70)	1,564	−0.427	−0.845
11	3,609	(3.38 3.83)	1,581	−0.577	−0.725
12	2,865	(2.63 3.10)	1750	0.112	−1,162
13	3,671	(3.46 3.88)	1,380	−0.686	−0.371
14	3,343	(3.10 3.59)	1887	−0.332	−1,158
15	2,415	(2.18 2.65)	1,692	0.570	−0.811
16	3,333	(3.10 3.57)	1710	−0.219	−1,113
17	3,894	(3.68 4.11)	1,419	−0.862	−0.326
18	3,860	(3.66 4.06)	1,299	−0.667	−0.632
19	2,860	(2.59 3.12)	2,207	0.118	−1,378
20	3,106	(2.85 3.36)	2008	−0.097	−1,274
21	2,802	(2.53 3.08)	2,371	0.168	−1,454
22	2058	(1.84 2.27)	1,427	0.862	−0.329
23	2,420	(2.17 2.67)	1934	0.550	−0.995
24	2,222	(2.00 2.44)	1,554	0.714	−0.528
25	3,213	(2.98 3.45)	1742	−0.270	−1,125
26	3,357	(3.13 3.59)	1,669	−0.378	−0.958
27	2,918	(2.66 3.18)	2,133	0.106	−1,371
28	3,242	(2.98 3.50)	2,145	−0.220	−1,347
29	1889	(1.69 2.09)	1,268	1,220	0.651
30	1966	(1.77 2.17)	1,260	1,014	0.191
31	3,159	(2.90 3.42)	2066	−0.164	−1,281
32	3,217	(2.98 3.46)	1803	−0.198	−1,115
33	2,483	(2.28 2.69)	1,361	0.435	−0.680
34	3,947	(3.73 4.16)	1,480	−0.998	−0.007
35	3,913	(3.70 4.13)	1,480	−0.997	0.025
36	4,150	(3.96 4.34)	1,132	−1,245	0.895
37	3,580	(3.33 3.83)	1915	−0.600	−0.894
38	3,536	(3.36 3.72)	1,012	−0.200	−0.477
39	3,831	(3.62 4.04)	1,377	−0.911	0.012
40	2,377	(2.13 2.62)	1897	0.593	−0.946

The average scores at the scale levels ranged from 1.88 to 4.15. The skewness and kurtosis of the items showed values within the limits, as per the perspective proposed by Kline (2011). The EFA process began by conducting Bartlett’s sphericity test (2208.6; *df* = 780; *p* < 0.001), which was significant, and the Kaiser-Meyer-Olkin (KMO) measure (KMO = 0.90), which was deemed adequate according to the literature (Howard, 2016; Rogers, 2022). Subsequently, permutation-based parallel analysis was applied, showing the retention of three factors. Therefore, EFA was performed with the number of factors fixed at three. Following the EFA execution, items #1, #2, #16, #18, #20, #21, #22, #28, #35, #39, #40 were excluded for having factor loadings below 0.30 or cross-loadings with the difference less than 0.20. Although item #26 (“During a performance, I feel an increase in muscle tension”) was statistically recommended (0.521), its grouping was loaded onto factor 1. However, this item would have made more sense if it had been grouped into factor 2 due to its theoretical similarity to item #25 (“Before a performance, I feel an increase in muscle tension”). Thus, we decided to exclude item #26. The remaining items achieved factor loadings above the recommended threshold (> 0.30), all factors were theoretically significant and plausible (Watkins, 2018), presenting a cumulative variance proportion of 52.64%. The 28-item model with three factors exhibited satisfactory fit indices (*χ2* = 478.050, *df* = 322; *χ2/df* = 1.48; CFI = 0.99; TLI = 0.98; RMSEA = 0.04). Table 3 presents the factorial model of the PoMPAS.

**Table 3:** Exploratory factor analysis results.

Item	Factor 1	Factor 2	Factor 3
#3	**0.339**		
#4	**0.556**		
#5	**0.832**		
#6	**0.637**		
#7	**0.594**		
#8	**0.705**		
#9	**0.573**		
#10	**0.628**		
#11	**0.842**		
#12	**0.676**		
#13	**0.600**		
#14	**0.795**		
#26	**0.521**		
#17		**0.831**	
#19		**0.689**	
#25		**0.465**	
#27		**0.461**	
#31		**0.914**	
#32		**0.440**	
#33		**0.617**	
#34		**0.749**	
#36		**0.561**	
#37		**0.511**	
#38	−0.586	**0.303**	
#15			**0.333**
#23			**0.594**
#24	0.316		**0.705**
#29			**0.797**
#30			**0.857**

We defined the three factors as: Behavioral/emotional factor (BEF – F1) composed of 13 items; Contextual/physiological factor (CPF – F2) composed of 11 items, and Cognitive factor (CF – F3) composed of 5 items. The measure of replicability of the factorial structure (H-index – Ferrando and Lorenzo-Seva, 2018) indicates that Factor 1 f(H-Observed: 0.96), Factor 2 (H-Observed: 0.93), and Factor 3 (H-Observed: 0.95) were above the recommended threshold. The three factors showed satisfactory indices (Factor 1: *α* = 0.91, CI 95%[0.90–0.93]; *ω* = 0.92, CI 95%[0.90–0.93]; Factor 2: *α* = 0.87, CI 95%[0.84–0.89]; *ω* = 0.88, CI 95%[0.85–0.90]; Factor 3: *α* = 0.84, CI 95%[0.81–0.87]; *ω* = 0.84, CI 95%[0.81–0.88]).

### 3.2 Confirmatory factor analysis

The three-factor model showed adequate fit measures (*χ2* = 866.39; *df* = 347; *p* < 0.001; *χ2/df* = 2.49; TLI = 0.98; CFI = 0.98; RMSEA = 0.09 CI 90% [0.08–0.09]); however, item #38 (“Even in contexts that cause me anxiety, I believe I will achieve a good performance”) presented a negative and low factorial load. In this sense, it was decided to exclude and check the fit indices as well as the factor loading of the remaining items. After excluding item #38, maintaining the factorial configuration, the model showed adequate fit measures (*χ2* = 870.23; *df* = 347; *p* < 0.001; *χ2/df* = 2.50; TLI = 0.98; CFI = 0.98; RMSEA = 0.09 CI 90% [0.08–0.09]), all items were statistically significant and loaded above 0.50. The CFA model was organized in the following manner: A Behavioral/Emotional Factor (BEF - F1) comprising of 12 items, a Contextual/Physiological Factor (CPF - F2) comprising of 10 items, and a Cognitive Factor (CF - F3) comprising of 5 items (as listed in Table 4), making a total of 27 items.

**Table 4:** Confirmatory factor analysis results.

			95% Confidence interval				
Factors	Items	Std. Est. (all)	Lower	Upper	Std. error	*z*-value	*p*
F1	3	0.61	0.57	0.65	0.02	30.78	<0.001
	4	0.74	0.70	0.77	0.02	41.93	<0.001
	5	0.67	0.64	0.71	0.02	38.24	<0.001
	6	0.70	0.67	0.74	0.02	40.08	<0.001
	7	0.62	0.58	0.65	0.02	33.58	<0.001
	8	0.77	0.74	0.81	0.02	44.62	<0.001
	9	0.78	0.75	0.82	0.02	48.07	<0.001
	10	0.86	0.83	0.90	0.02	54.76	<0.001
	11	0.79	0.76	0.82	0.02	48.84	<0.001
	12	0.68	0.65	0.72	0.02	38.22	<0.001
	13	0.64	0.61	0.68	0.02	36.16	<0.001
	14	0.66	0.63	0.70	0.02	37.05	<0.001
F2	17	0.72	0.69	0.76	0.02	38.41	<0.001
	19	0.68	0.65	0.72	0.02	37.66	<0.001
	25	0.69	0.65	0.72	0.02	37.41	<0.001
	27	0.54	0.50	0.58	0.02	26.53	<0.001
	31	0.81	0.77	0.84	0.02	48.57	<0.001
	32	0.76	0.73	0.80	0.02	44.25	<0.001
	33	0.64	0.61	0.68	0.02	34.46	<0.001
	34	0.87	0.84	0.90	0.02	49.61	<0.001
	36	0.69	0.65	0.73	0.02	36.74	<0.001
	37	0.76	0.73	0.80	0.02	42.87	<0.001
F3	15	0.77	0.73	0.80	0.02	37.66	<0.001
	23	0.94	0.90	0.97	0.02	53.71	<0.001
	24	0.90	0.86	0.93	0.02	52.47	<0.001
	29	0.84	0.80	0.88	0.02	41.36	<0.001
	30	0.83	0.79	0.87	0.02	41.54	<0.001

It is worth mentioning that most correlations between items and factors proved to be strong (0.34 to 0.64). Each dimension evidenced an acceptable internal consistency (Factor 1: α = 0.90, CI 95%[0.88–0.92]; ω = 0.90, CI 95%[0.88–0.92]; Factor 2: α = 0.88, CI 95%[0.85–0.90]; ω = 0.88, CI 95%[0.86–0.91]; Factor 3: α = 0.81, CI 95%[0.77–0.85]; ω = 0.81, CI 95%[0.77–0.85]), demonstrating good reliability. The values of the AVE (Average Variance Extracted) were adequate (Factor 1: 0.52; Factor 2: 0.52; Factor 3: 0.62).

## 4 Discussion

This research aimed to determine if the Portuguese Music Performance Anxiety Scale (PoMPAS) – in Portuguese: “Escala Portuguesa de Avaliação da Ansiedade na Performance Musical (EPAAPM)” – is a valid and reliable measure for the context of higher music education in Portugal.

The interpretation process began by conducting EFA with the number of factors identified, as well as assessing the KMO and Bartlett’s sphericity indices (Lloret-Segura et al., 2014; Watkins, 2018; Rogers, 2022). Current guidelines with recommendations for conducting EFA describe the superior performance of parallel analysis in ordinal data compared to other factor extraction techniques (Schreiber, 2021; Rogers, 2022). Therefore, for the current study, we opted to use Parallel Analysis, which indicated the extraction of two factors. In this sense, we tested the structure regarding the feasibility of three factors. Considering both the conceptual relationship of the items and the factor loading in each obtained factor, the three-factor model has good psychometric properties.

In this exploratory model, Factor 1 captured the construct related to behavioral/emotional, while Factor 2 referred to contextual/physiological, and Factor 3 related to cognitive. The three factors were well-defined and exhibited high replicability [Factor 1: (H-Observed: 0.96), Factor 2: (H-Observed: 0.93), Factor 3: (H-Observed: 0.95)]. Additionally, the exploratory model demonstrated a good fit based on satisfactory fit indices.

Following the exploratory factor analysis (EFA) outlined previously, a confirmatory factor analysis (CFA) was conducted to further validate the factorial structure of the PoMPAS. The CFA aimed to confirm the fit of the three-factor model (BEF – F1, CPF – F2, and CF – F3), as identified in the EFA. After excluding item #38, the confirmatory factor analysis showed adequate fit indices in the multifactorial solution of three first-order factors. Furthermore, the CFA demonstrated that a model with three dimensions was justified in theory and practice. It is worth mentioning that, in a complex model (i.e., with various parameters to be estimated), the *χ2* result was not considered as a criterion for discarding the model (Kyriazos, 2018). The reliability, assessed using both Cronbach’s alpha and McDonald’s omega, was satisfactory across exploratory and confirmatory analyses. Notably, McDonald’s omega is highlighted as a preferred index over Cronbach’s alpha due to its consideration of the individual importance of each item within the construct, as determined by their factor loadings (McNeish, 2018; Schreiber, 2021).

Comparing the PoMPAS with the seven MPA scales described above highlights both similarities and differences. While the PoMPAS assesses behavioral/emotional, contextual/physiological, and cognitive dimensions associated with MPA, the MPAS (Wolfe, 1989) measures MPA through cognitive and emotional components, seeking to understand adaptive and maladaptive anxiety. The MPAQ (Lehrer et al., 1990) emphasizes coping strategies, judgmental attitudes/thoughts about performance, concern about the impact of anxiety and worry about the reaction of others, oneself and the audience. Fehm and Schmidt’s (2006) PAQ, on the other hand, assesses cognitive and somatic feelings and seeks to understand feelings of performance anxiety and coping strategies. The K-MPAI (Kenny, 2009) assesses proximal somatic anxiety and performance worries, negative cognitions, psychological vulnerability, parental empathy, memory, generational transmission of anxiety, apprehension and biological vulnerability. The PASMS (Cırakoğlu and Şentürk, 2013) measures stage fright, avoidance and symptoms of MPA. The MPAS (Sheriff and Yoong, 2015) evaluates the frequency and intensity of situational factors, occurrence, and cognitive, affective, behavioral and somatic manifestations. The M-MPAS (Mazzarolo and Schubert, 2022) assesses MPA’s global frequency and intensity and the negative impact on future music performances.

After checking the differences between the scales, we can verify that each has specific assets for measuring MPA. However, none of the instruments shown takes into account the context of the performance situation, a crucial factor that limits the understanding of MPA, particularly in higher education, the primary focus of this research.

Authors such as Papageorgi et al. (2010), Zarza et al. (2016a), and Casanova et al. (2018) have highlighted the influence of the performance situation context on MPA levels. This factor, which is often overlooked, is a key strength of this study. By addressing this influence, the study not only enhances our understanding of MPA but also contributes to the development of more effective MPA measurement tools.

The PoMPAS is designed to understand, confirm, and identify the global characteristics of students’ MPA in that context. Moreover, the PoMPAS scale stands out due to its emphasis on assessing MPA “before” and “during” performance, setting it apart from other scales. This perspective has been mentioned by music students in the Portuguese context (Barros et al., 2024), highlighting that the psychophysiological sensations of MPA are predominantly experienced “before” and “during” (and to a lesser extent, “after”) the performance. This insight opens avenues for developing tailored strategies to alleviate MPA in these specific moments, while considering the unique profile of each student. The PoMPAS may have a positive impact in this field since it broadens the understanding and assessment of MPA and, consequently, addresses limitations of other scales.

According to Rocha Zaidhaft and Ortega (2021), the cultural context has become increasingly relevant for health actions. Kirmayer (2014a,b) highlights that cultural contexts are diverse, encompassing not only spatial but also temporal and historical dimensions within a society. The cultural context plays a crucial role in shaping knowledge and identity, providing significance and direction to human life, and, as a result, affecting aspects related to mental health such as anxiety and depression. This implies that the context has an impact not only on how MPA is experienced but also on how it is perceived and addressed by higher music education institutions.

Barros’ systematic review (Barros et al., 2022) showed that both the contextual factor of the performance situation and the behavioral factor are predictors of MPA. However, in this study, the contextual dimension was associated with the physiological factor (CPF) and the behavioral dimension was associated with the emotional factor (BEF) for better adjustment indices, suggesting two new variables to consider in future research. Furthermore, these variables can also support decision-making to implement specific interventions involving music students.

This study has some limitations that can be addressed by future research. Since the PoMPAS (Appendix 1) is a self-report questionnaire, its limitations are inherent to the type of instrument itself (it only assesses MPA globally). The sample in this study was convenience-based, which hinders generalizations. Furthermore, other questionnaires that could test convergent and divergent validity were not applied, which may be the main shortcoming of this study. Thus, we suggest that future studies include this validation with different scales, seeking to identify possible weaknesses. Despite this, the PoMPAS has proven to be a reliable tool with good psychometric qualities, developed and validated to measure MPA in the context of higher music education in Portugal.

## Data availability statement

The raw data supporting the conclusions of this article will be made available by the authors, without undue reservation.

## Ethics statement

The studies involving humans were approved by the Ethics and Deontology Board of the University of Aveiro (Certificate 02-CED/2020). The studies were conducted in accordance with the local legislation and institutional requirements. The participants provided their written informed consent to participate in this study.

## Author contributions

SB: Conceptualization, Investigation, Writing – original draft, Writing – review & editing, Methodology, Data curation. AF: Data curation, Formal analysis, Software, Validation, Writing – review & editing. HM: Methodology, Supervision, Writing – review & editing. AP: Supervision, Writing – review & editing.
