# Supplementary material for: Music performance anxiety: development and validation of the Portuguese music performance anxiety scale
Source: Front Psychol. 2024 Jul 12;15:1436216. doi: 10.3389/fpsyg.2024.1436216 (PMC11276725; doi:10.3389/fpsyg.2024.1436216)
Supplement: Supplementary file 1 [file Data_Sheet_1.docx]

Music performance anxiety: development and validation of the Portuguese music performance anxiety scale (PoMPAS)

Samuel Barros*, Alex França, Helena Marinho, Anabela Pereira^3^

*** Correspondence:**

samuelbarros@ua.pt

**Appendix 1**

| **Portuguese music performance anxiety scale (PoMPAS)**  **Escala Portuguesa de Avaliação da Ansiedade na Performance Musical (EPAAPM)**  Original version with English translation | | | | | |
| --- | --- | --- | --- | --- | --- |
| **Mark your level of agreement for each item: 1 = I completely disagree and 5 = I completely agree.**  **Marque o seu nível de concordância em relação a cada item. Note que 1 = discorda completamente e 5 = concorda completamente.** | **1** | **2** | **3** | **4** | **5** |
| **Behavioural/emotional factor – Fator comportamental/emocional** |  |  |  |  |  |
| 1. (3). Before a performance, I create expectations about how it will go. / Antes de uma performance crio expectativas sobre como esta vai correr. |  |  |  |  |  |
| 2. (4). Regardless of how well prepared I am, I feel anxious and make mistakes or technical faults during a performance. / Durante uma performance, independentemente de estar bem preparado, sinto ansiedade e cometo erros ou falhas técnicas. |  |  |  |  |  |
| 3. (5). Making mistakes during a performance compromises my capacity to continue. / Cometer erros durante uma performance compromete a minha capacidade de continuar. |  |  |  |  |  |
| 4. (6). Before a performance, I worry about the negative audience reaction. / Antes de uma performance preocupo-me com a reação negativa do público. |  |  |  |  |  |
| 5. (7). Before a performance, I worried that a bad performance could ruin my career. / Antes de uma performance preocupa-me que uma má performance possa arruinar a minha carreira. |  |  |  |  |  |
| 6. (8). During a performance, I question my capacity to make it to the end. / Durante uma performance questiono a minha capacidade de chegar ao fim. |  |  |  |  |  |
| 7. (9). Before a performance, I have negative thoughts. / Antes de uma performance tenho pensamentos negativos. |  |  |  |  |  |
| 8. (10). The worry and nervousness before a performance interferes with my focus and concentration. / A preocupação e o nervosismo antes de uma performance interferem no meu foco e concentração. |  |  |  |  |  |
| 9. (11). The worry and nervousness during a performance interferes with my focus and concentration. / A preocupação e o nervosismo durante uma performance interferem no meu foco e concentração. |  |  |  |  |  |
| 10. (12). The memory of previous performances interferes negatively when I play in public. / A recordação de performances anteriores interfere negativamente quando toco em público. |  |  |  |  |  |
| 11. (13). Before a performance, I worry about my performance skills. / Antes de uma performance preocupo-me com as minhas competências performativas. |  |  |  |  |  |
| 12. (14). During a performance, I worry about my performance skills. / Durante uma performance preocupo-me com as minhas competências performativas. |  |  |  |  |  |
| **Contextual/Physiological factor – Fator contextual/fisiológico** |  |  |  |  |  |
| 13. (17). Before a performance, I feel my heart rate accelerating. / Antes de uma performance sinto uma aceleração da frequência cardíaca. |  |  |  |  |  |
| 14. (19). Before a performance, I feel shaky. / Antes de uma performance sinto tremores. |  |  |  |  |  |
| 15. (25). Before a performance, I feel an increase in muscle tension. / Antes de uma performance sinto um aumento da tensão muscular. |  |  |  |  |  |
| 16. (27). Before a performance, my mouth feels dry. / Antes de uma performance sinto a boca seca. |  |  |  |  |  |
| 17. (31). Before a performance, I feel my breathing is altered. / Antes de uma performance sinto a respiração alterada. |  |  |  |  |  |
| 18. (32). During a performance, I feel my breathing is altered. / Durante uma performance sinto a respiração alterada. |  |  |  |  |  |
| 19. (33). Before a group performance, I feel anxiety. / Antes de uma performance em grupo sinto ansiedade. |  |  |  |  |  |
| 20. (34). Before a solo performance, I feel anxiety. / Antes de uma performance a solo sinto ansiedade. |  |  |  |  |  |
| 21. (36). I feel anxiety when I play in competitions or auditions for orchestra. / Sinto ansiedade quando toco em concursos ou audições para orquestra. |  |  |  |  |  |
| 22. (37). The formalities of the performance context cause me anxiety. / As formalidades do contexto da performance causam-me ansiedade. |  |  |  |  |  |
| **Cognitive factor – Fator cognitivo** |  |  |  |  |  |
| 23. (15). In the preparation phase of the performance, I feel fear or panic. / Na fase de preparação de uma performance sinto medo ou pânico. |  |  |  |  |  |
| 24. (23). Before a performance, I feel sensations of threat or panic. / Antes de uma performance sinto sensações de ameaça ou pânico. |  |  |  |  |  |
| 25. (24). During a performance, I feel sensations of threat or panic. / Durante uma performance sinto sensações de ameaça ou pânico. |  |  |  |  |  |
| 26. (29). Before a performance, I feel a lack of strength. / Antes de uma performance sinto falta de força. |  |  |  |  |  |
| 27. (30). During a performance, I feel a lack of strength. / Durante uma performance sinto falta de força. |  |  |  |  |  |
